# Essential fatty acids and their metabolites could function as endogenous HMG-CoA reductase and ACE enzyme inhibitors, anti-arrhythmic, anti-hypertensive, anti-atherosclerotic, anti-inflammatory, cytoprotective, and cardioprotective molecules

**DOI:** 10.1186/1476-511X-7-37

**Published:** 2008-10-15

**Authors:** Undurti N Das

**Affiliations:** 1 UND Life Sciences, 13800 Fairhill Road, #321, Shaker Heights, OH 44120, USA; 2 Department of Medicine, Bharati Vidyapeeth University Medical College, Pune, India

## Abstract

Lowering plasma low density lipoprotein-cholesterol (LDL-C), blood pressure, homocysteine, and preventing platelet aggregation using a combination of a statin, three blood pressure lowering drugs such as a thiazide, a β blocker, and an angiotensin converting enzyme (ACE) inhibitor each at half standard dose; folic acid; and aspirin-called as polypill- was estimated to reduce cardiovascular events by ~80%. Essential fatty acids (EFAs) and their long-chain metabolites: γ-linolenic acid (GLA), dihomo-GLA (DGLA), arachidonic acid, eicosapentaenoic acid (EPA), and docosahexaenoic acid (DHA) and other products such as prostaglandins E_1 _(PGE_1_), prostacyclin (PGI_2_), PGI_3_, lipoxins (LXs), resolvins, protectins including neuroprotectin D_1 _(NPD_1_) prevent platelet aggregation, lower blood pressure, have anti-arrhythmic action, reduce LDL-C, ameliorate the adverse actions of homocysteine, show anti-inflammatory actions, activate telomerase, and have cytoprotective properties. Thus, EFAs and their metabolites show all the classic actions expected of the "polypill". Unlike the proposed "polypill", EFAs are endogenous molecules present in almost all tissues, have no significant or few side effects, can be taken orally for long periods of time even by pregnant women, lactating mothers, and infants, children, and adults; and have been known to reduce the incidence cardiovascular diseases including stroke. In addition, various EFAs and their long-chain metabolites not only enhance nitric oxide generation but also react with nitric oxide to yield their respective nitroalkene derivatives that produce vascular relaxation, inhibit neutrophil degranulation and superoxide formation, inhibit platelet activation, and possess PPAR-γ ligand activity and release NO, thus prevent platelet aggregation, thrombus formation, atherosclerosis, and cardiovascular diseases. Based on these evidences, I propose that a rational combination of ω-3 and ω-6 fatty acids and the co-factors that are necessary for their appropriate action/metabolism is as beneficial as that of the combined use of a statin, thiazide, a β blocker, and an angiotensin converting enzyme (ACE) inhibitor, folic acid, and aspirin. Furthermore, appropriate combination of ω-3 and ω-6 fatty acids may even show additional benefits in the form of protection from depression, schizophrenia, Alzheimer's disease, and enhances cognitive function; and serve as endogenous anti-inflammatory molecules; and could be administered from childhood for life long.

## Introduction

Cardiovascular diseases (CVD) are responsible for significant morbidity and mortality throughout the world. Studies revealed that smoking cessation, β-blockers, anti-platelet agents, angiotensin converting enzyme (ACE) inhibitors, and lipid lowering agents such as statins, each reduce the risk of vascular events to a moderate but important degree [1-9]. In addition, observational studies suggested lower rates of fractures and dementia with statins, and lower rates of cataracts with anti-oxidant vitamins, though these observations need to be confirmed by randomised trials [9]. The results of the MRC/BHF-HPS study led to the suggestion that using a combination of aspirin, β-blockers, statins, and ACE inhibitors could prevent about two-thirds to three-quarters of future vascular events [10]. It was suggested that a combination pill (called as "polypill") consisting of atorvastatin 10 mg or simvastatin 40 mg; three blood pressure lowering drugs such as a thiazide, a β-blocker, and an ACE inhibitor, each at half standard dose; folic acid 0.8 mg; and aspirin 75 mg could reduce coronary heart disease (CHD) events by 88% (95% confidence interval 84% to 91%) and stroke by 80% (71% to 87%), and if such a combination pill is taken from age 55 years of age, at least one third of people taking it, would on an average add about 11 years of life free from an CHD event or stroke [11].

Further support to the concept of polypill for the prevention of primary and secondary cardiovascular diseases proposed by Wald and Law [11] is provided by the work of Hippisley-Cox and Coupland [12] who examined the individual and combined effects of three of the polypill ingredients-statins, aspirin, and blood pressure lowering drugs. Their analysis of 11330 patients with CHD showed that all cause mortality is lower in those taking two or three drugs compared with those taking single agents. These findings are consistent with previous studies [13,14] that showed that a combination of two drugs-aspirin and statin-is superior to either drug alone in the secondary prevention of CHD. However, it was also noted that synergistic effects are seen with two, but not three or four, drug combinations in secondary prevention of CHD.

But concerns have been raised about the adverse effects of such a polypill. For instance, β blockers are unsuitable for subjects with bronchial asthma, and some are intolerant to aspirin and develop significant gastrointestinal side effects. It may be necessary to closely monitor to detect serious adverse effects of statins, and renal failure due to ACE inhibitors and angiotensin-II receptor antagonists. Furthermore, the efficacy of aspirin in men is established [15], but its efficacy in women is not certain [16]. A recent study showed that 2.5 mg of folic acid (the proposed polypill dosage is 0.8 mg/day) was not associated with a reduction in stroke, coronary events, and death in patients who previously had a cerebral infarction despite a moderate reduction of total homocysteine during the 2 years of follow-up [17]. Another major concern about the primary prevention strategy of the "polypill" is related to the possibility that it might lead to medicalising of the population since, it is highly cost effective to treat individuals at high risk compared to those at lower risk that is much more expensive to treat in terms of gain in quality adjusted life years. Hence, it will be worthwhile to look for alternatives to "polypill" that is less expensive, more acceptable, less likely to cause side effects and does not lead to medicalising the population. I propose that a rational combination of ω-3 and ω-6 fatty acids may, in fact, be more beneficial compared to the "polypill" in the primary and secondary prevention of cardiovascular diseases.

### Metabolism of essential fatty acids

Essential fatty acids (EFAs) are essential for survival and they cannot be synthesized in the body and hence, have to be obtained in our diet and thus, are essential [18-21]. There are two types of naturally occurring EFAs in the body, the ω-6 series derived from linoleic acid (LA, 18:2) and the ω-3 series derived from α-linolenic acid (ALA, 18:3). Both ω-6 and ω-3 series are metabolized by the same set of enzymes to their respective long-chain metabolites. While some of the functions of EFAs require their conversion to eicosanoids and other products, in majority of the instances the fatty acids themselves are active.

**Figure 1:**
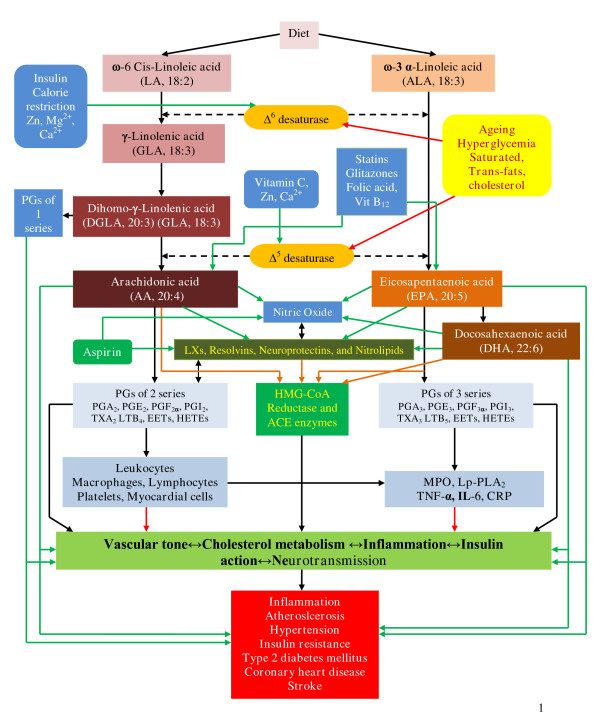
**Metabolism of essential fatty acids**. Prostaglandins of 3 series are less pro-inflammatory compared to prostaglandins of 2 series. Resolvins are formed from both EPA and DHA and are known to have anti-inflammatory actions and participate in the resolution of inflammation. EPA can be converted to DHA. DHA can be retroconverted to EPA. It is estimated that about 30–40% of DHA can be retroconverted to EPA. The biochemical and/or clinical significance of this retroconversion of DHA to EPA are not known. ____ Indicates beneficial action in the form of increase in the synthesis, action, or remission of disease process. ____ Indicates decrease in the synthesis, action or enhancement of pathological process. ____ Indicates inhibition of HMG-CoA and ACE enzymes by PUFAs/EFAs.

LA is converted to γ-linolenic acid (GLA, 18:3, n-6) by the action of the enzyme Δ^6 ^desaturase (d-6-d) and GLA is elongated to form dihomo-GLA (DGLA, 20:3, n-6), the precursor of the 1 series of prostaglandins (PGs). DGLA can also be converted to arachidonic acid (AA, 20:4, n-6) by the action of the enzyme Δ^5 ^desaturase (d-5-d). AA forms the precursor of 2 series of prostaglandins, thromboxanes and the 4 series of leukotrienes. ALA is converted to eicosapentaenoic acid (EPA, 20:5, n-3) by d-6-d and d-5-d. EPA forms the precursor of the 3 series of prostaglandins and the 5 series of leukotrienes (see Figure 1 for the metabolism of EFAs). AA and EPA give rise to their respective hydroxy acids, which, in turn, are converted to respective leukotrienes (LTs). In addition, AA, EPA, and DHA form precursor to anti-inflammatory compounds lipoxins, resolvins, and protectins (neuroprotectin D_1 _is one such compound derived from DHA) [22-27]. PGs, LTs, lipoxins (LXs), and resolvins are highly active, modulate inflammation, and are involved in several physiological and pathological processes [18]. In general, the term "EFAs" is used to refer to all unsaturated fatty acids: LA, GLA, DGLA, AA, ALA, EPA, and DHA; and the term polyunsaturated fatty acids (PUFAs) refer to GLA, DGLA, AA, EPA, and DHA. Although the terms EFAs and PUFAs are used interchangeably, it should be understood that all EFAs are PUFAs but all PUFAs are not EFAs. Only LA and ALA qualify to be EFAs; whereas GLA, DGLA, AA, EPA, and DHA are PUFAs. On the other hand, LA, GLA, DGLA, AA, ALA, EPA, and DHA are also called as LCPUFAs (long-chain polyunsaturated fatty acids). Many of the functions of EFAs are also brought about by PUFAs and EFA-deficiency states can be corrected to a large extent by PUFAs that suggests that PUFAs are "functional EFAs". Hence, in general, many authors use the terms EFAs and PUFAs interchangeably. This convention is followed in the present discussion.

### Dietary sources of EFAs

EFAs: LA and ALA are present in human diet in abundant amounts and hence, EFA-deficiency is uncommon. In certain specific conditions such as total parenteral nutrition (TPN) and severe malabsorption occasionally EFA deficiency could be seen. The present TPN solutions contain adequate amounts of EFAs. The manifestations of EFA deficiency include: dry and scaly skin, hepatospleenomegaly, immunodeficiency, inappropriate water loss through the skin, dehydration, scalp dermatitis, alopecia, and depigmentation of hair. EFAs are widely distributed in normal human diet. The main dietary sources of EFAs are as follows.

Human breast milk is rich in all types of PUFAs [18] that explains why breast-fed children are healthier compared to bottle-fed. LA and ALA are present in significant amounts in dairy products, organ meats such as liver, and many vegetable oils such as sunflower, safflower, corn and soy. GLA is present in evening primrose oil at concentrations of 7–14% of total fatty acids; in borage seed oil it is 20–27%; and in black currant seed oil at 15–20%. GLA is also found in some fungal sources [18]. DGLA is found in liver, testes, adrenals, and kidneys. AA is present in meat, egg yolks, some seaweeds, and some shrimps. Average daily intake of AA is estimated to be ~100–200 mg/day that accounts for the total daily production of various PGs. EPA and DHA are present mainly in marine fish. Cow's milk contains very small amounts of GLA, DGLA and AA.

EFAs/PUFAs are unstable due to the presence of 2 or more double bonds in their structure. Substantial loss of EFAs/PUFAs occurs during food processing and hydrogenation of oils. Exposure to high temperatures and hydrogenation process causes denaturation of EFAs/PUFAs and their conversion to trans-fats that are harmful to the body [18]. Human diet was rich in ω-3 fatty acids in the early humans. But with the progress in industrialization and development of fast foods, the content of ω-3 fatty acids in human diet dwindled, whereas that of ω-6 fatty acids increased. The ratio of ω-3 to ω-6 fatty acids in the diet of early humans was >1, whereas this ratio is now about 10:1 to 20–25:1. It is recommended that the ratio between ω-3 to ω-6 fatty acids in the diet should be about 1 or >1 and preferably 2–3:1. This fall in the intake of ω-3 fatty acids, especially EPA and DHA in the last 50 years is believed to be responsible for the increasing incidence of atherosclerosis, CHD, hypertension, metabolic syndrome X, obesity, collagen vascular diseases and possibly, cancer. This is supported by the observation that increasing dietary α-linolenate/linoleate balance affected the ω-3/ω-6 ratio of brain phospholipid acyl chains and produced changes in general behavior as well as changes in sensitivities to drugs known to affect behavior, influenced LTs formation in polymorpho-nuclear leukocytes from AA and EPA and release of histamine from mast cells that could alter the severity of allergic and inflammatory responses. This increase in ω-3 fatty acids also resulted in an increased mean survival time of SHR-SP (spontaneously hypertensive-stroke prone) rats by lowering blood pressure and platelet aggregability, produced significant changes in Na^+^-K^+^-ATPase activity, and altered collagen-induced platelet aggregation and serotonin release in experimental animals. These results suggest that enhanced intake of ω-3 fatty acids is of significant benefit in various diseases.

### Factors influencing the metabolism of EFAs

Dietary LA and ALA are metabolized by the same set of Δ^6 ^and Δ^5 ^desaturases and elongases to their respective metabolites (see Figure 1). These 2 fatty acids compete with one another for the same set of enzymes and Δ^6 ^and Δ^5 ^desaturases prefer ω-3 to ω-6. Oleic acid (OA, ω-9) that is not an EFA is also metabolized by the same desaturases. But, in view of the preference of these enzymes to LA and ALA, under normal physiological conditions, the metabolites of ω-9 are formed only in trivial amounts. Hence, presence of significant amounts of 20:3 ω-9, a metabolite of OA, in the cells and plasma indicates EFA deficiency that is utilized to detect the presence of EFA deficiency in patients, experimental animals and in vitro studies.

Of several factors that influence the activities of desaturases and elongases [18], saturated fats, cholesterol, trans-fatty acids, alcohol, adrenaline, and glucocorticoids inhibit Δ^6 ^and Δ^5 ^desaturases. Pyridoxine, zinc, and magnesium are necessary co-factors for normal Δ^6 ^desaturase activity. Insulin activates Δ^6 ^desaturase whereas diabetics have reduced Δ^6 ^desaturase activity. The activity of Δ^6 ^desaturase falls with age. Oncogenic viruses and radiation inhibit Δ^6 ^desaturase. Total fasting, protein deficiency, glucose rich diets reduce the activity of Δ^6 ^desaturase. A fat- free diet and partial caloric restriction enhances Δ^6 ^desaturase. Activities of Δ^6 ^and Δ^5 ^desaturases are decreased in diabetes mellitus, hypertension, hyperlipidemia, and metabolic syndrome X. Trans-fats, saturated fatty acids, and cholesterol interfere with EFA metabolism and promote inflammation, atherosclerosis and coronary heart disease (CHD) [18]. This implies that trans-fats, saturated fats, and cholesterol have pro-inflammatory actions whereas EFAs and PUFAs possess anti-inflammatory properties. This explains why trans-fats, saturated fats, and cholesterol are pro-atherogenic, whereas EFAs/PUFAs, especially ω-3 fatty acids are anti-atherogenic. The ability of trans-fats, saturated fats, and cholesterol to interfere with the formation of AA, EPA, and DHA from dietary LA and ALA could lead to decreased formation of LXs, resolvins, PGI_2 _(prostacyclin), PGI_3_, and other beneficial eicosanoids that prevent platelet aggregation, leukocyte chemotaxis and activation. LXs and resolvins decrease the formation of pro-inflammatory cytokines, and produce vasodilatation, events that prevent or arrest atheroslcerosis. In contrast, trans-fats, saturated fats, and cholesterol may directly activate leukocytes, induce the generation of free radicals and enhance the production and release of pro-inflammatory cytokines that facilitate atherosclerosis [18]. Trans-fats, saturated fats, and cholesterol directly activate leukocytes and macrophages to induce them to produce free radicals and pro-inflammatory cytokines:

IL-6, TNF-α, IL-1, IL-2, and MIF (macrophage migration inhibitory factor). This action of trans-fats, saturated fats, and cholesterol is in addition to their ability to suppress metabolism of EFAs to their respective long-chain metabolites. It is possible that trans-fats, saturated fats, and cholesterol may also have the ability to inhibit the formation of LXs, resolvins, PGI_2_, and PGI_3_. These studies suggest that EFAs, especially EPA and DHA are cytoprotective to endothelial cells, whereas trans-fats, saturated fats, and cholesterol produce endothelial dysfunction. AA, EPA, and DHA augment nitric oxide generation from endothelial cells [18] and thus help in the prevention of endothelial dysfunction. In contrast, trans-fats, saturated fats, and cholesterol produce endothelial dysfunction and thus, inhibit eNO production. Furthermore, NO quenches superoxide anion and thus, prevents the cytotoxic action of superoxide anion and protects endothelial cells from free radical-induced damage. This implies that for endothelial cells to be healthy they need adequate amounts of AA, EPA, and DHA so that they can generate physiological amounts of eNO not only to prevent pathological platelet aggregation and atherosclerosis but also to protect themselves from the cytotoxic actions of free radicals.

Furthermore, NO reacts with PUFAs to yield their respective nitroalkene derivatives that can be detected in plasma. These nitroalkene derivatives, termed as nitrolipids, produce vascular relaxation, inhibit neutrophil degranulation and superoxide formation, and inhibit platelet activation, and show anti-atherosclerotic properties [18]. Thus, there appears to be a close interaction between EFAs and their products and trans-fats, saturated fats, and cholesterol with regard to the ability of endothelial cells to produce PGI_2_, PGI_3_, NO, and other anti-atheroslcerotic and beneficial molecules.

## Actions of PUFAs that qualify them to be the endogenous HMG-CoA reductase and ACE enzyme inhibitors, anti-arrhythmic, anti-hypertensive, anti-atherosclerotic, anti-inflammatory, cytoprotective, and cardioprotective molecules

### Action on HMG-CoA reductase

PUFAs are potent inhibitors of HMG-CoA reductase enzyme and similar to statins are useful in the treatment of hyperlipidemias [28-33]. Statins enhance plasma AA levels and decrease the ratio of EPA to AA significantly [29,30], and enhance the formation of prostacyclin (PGI_2_) [32]. In fact, statins and PUFAs have many overlap actions such as inhibition of IL-6 and TNF-α production and NF-κB activation, increasing the synthesis of endothelial nitric oxide (eNO); and both are anti-inflammatory in nature [18,33-37]. In addition, a close interaction exists between NO and COX enzymes attesting to the fact that statins, PUFAs, and NO have positive and negative influences among themselves [38]. Both statins and PUFAs are useful in atherosclerosis, coronary heart disease, osteoporosis, stroke, Alzheimer's disease, and inflammatory conditions such as lupus [18,39-52]. These evidences suggest that PUFAs mediate many, if not all, actions of statins [33] and this could be one mechanism by which they lower cholesterol levels. Recent studies revealed that statins augment concentrations of LXs in the heart [53,54] lending support to this concept. Furthermore, when a combination of statins and PUFAs are given together a synergistic beneficial effect was seen in patients with combined hyperlipemia [55-58]. But statins cannot be given during pregnancy, whereas PUFAs have been recommended during pregnancy, lactation and infancy to improve brain growth and development, and cognitive function [18-21], [59-67], though some studies did not show improvement in cognition [68,69]. Nevertheless, these studies reported that supplementation of PUFAs to pregnant and lactating women and infants is safe and without any side effects.

### PUFAs modulate renin formation, ACE activity and endothelial nitric oxide generation

Angiotensin converting enzyme (ACE) inhibitors are useful to lower blood pressure [11]. Renin, a proteolytic enzyme, is produced and stored in the granules of the juxtaglomerular cells in the kidney. Renin acts on angiotensinogen (a circulating α_2 _globulin made in the liver) to form the decapeptide angiotensin-I (Ang-I). Ang-I is transformed by ACE to angiotensin-II (Ang-II). Ang-II controls blood pressure and regulates body fluid volume by modulating renin-angiotensin-aldosterone system. ACE is present in the uterus, placenta, vascular tissue, heart, brain, adrenal cortex and kidney, leukocytes, alveolar macrophages, peripheral monocytes, neuronal cells and epididymal cells [70]. Angiotensin receptors are AT1 and AT2. AT1 exists as two subtypes α and β. Actions of Ang-II are mediated by the AT1 receptor. Angiotensinases, present in several tissues, destroy Ang-II (half-life approximately 1 minute), while the half-life of renin is about 10 to 20 minutes. In addition to circulating renin-angiotensin, many tissues have a local renin-angiotensin system and thus, have the ability to produce Ang-II. Locally generated Ang-II is involved in the modulation of growth and function of many tissues including vascular smooth muscle.

Linseed oil (which contains approximately: 19% oleic acid, 24% LA, and 47% ALA) fed experimental animals showed significantly lower renal venous renin secretion rates relative to the saturated fat-fed control group. Dietary enrichment with 20 energy % PUFA lowered renin secretion by a prostaglandin-independent mechanism that might have contributed to the lower blood pressures observed compared with the saturated fat-fed control group [71]. Dietary supplementation with 3 gm/day of LA and 32 mg/day of GLA to pregnant and non-pregnant subjects showed that the diastolic pressor response to angiotensin-II was significantly less in the pregnant subjects compared to the non-pregnant subjects, suggesting that PUFAs modulate vascular tissue responses to angiotensin-II. This decreased response to angiotensin-II could be due to increased formation of PGE_1 _and PGI_2 _in fatty acid supplemented pregnant subjects [72,73]. In a model of hypertension induced by continuous infusion of angiotensin-II in the rat, subcutaneous administration of LA and EPA and DHA were found to be equally potent in reducing, by half, the rise in systolic blood pressure induced by angiotensin-II and these anti-hypertensive effects were not accompanied by any changes in the renal synthesis of PGI_2 _or PGE_2_. Furthermore, indomethacin, a potent inhibitor of PGI_2 _but not of PGE_2 _synthesis could only partially neutralize the anti-hypertensive effects of LA and EPA/DHA, emphasizing that the anti-hypertensive effects are independent of the PG system [74]. This idea is reinforced by the observation that LA and AA inhibit renin, and thus, the overall activity of the renin-angiotensin-aldosterone cascade could be modified by alterations of plasma fatty acid concentrations [75,76], though in two-kidney, one-dip hypertensive animals, pretreatment with indomethacin did not alter hypotensive response to LA despite the fact that LA infusion lowered blood pressure in high renin but not in low renin states and the reduction in blood pressure was not related to inhibition circulating renin or to alterations of endogenous PG biosynthesis [77]. These evidences suggest that PUFAs have modulatory influence on renin secretion and action and yet times independent of both renin secretion and PG formation. Since the anti-hypertensive actions of PUFAs seem to be independent of formation of PGs, it is likely that fatty acids themselves are able to bring about this action and/or converted to form lipoxins, resolvins and protectins that have anti-inflammatory action and also possibly, anti-hypertensive action. But this proposal needs to be verified and confirmed. Yet another possibility is that the PUFAs supplemented are incorporated into the cell membrane phospholipid fraction that is able to enhance the synthesis of eNO that is a potent vasodilator and platelet anti-aggregator.

Previously, I showed that PUFAs inhibit leukocyte ACE activity [78]. Of all the fatty acids tested, EPA was the most effective (EPA > ALA > DHA > GLA > LA > AA), whereas AA was the least effective when their ability to inhibit purified ACE activity was tested. DHA and EPA were the most effective fatty acids in inhibiting the leukocyte ACE activity (EPA > DHA > ALA = AA > LA > GLA). On the other hand, PGs (PGE_1_, PGE_2_, PGI_2 _and PGF_2α_), and free radicals: superoxide anion, hydrogen peroxide, and hydroxyl radical showed marginal (~20%) inhibitory action on ACE activity. In contrast, NO (nitric oxide) showed powerful inhibitory action on ACE activity [78], whereas PUFAs enhanced endothelial nitric oxide (eNO) generation [36,47,79]. The effects of PUFAs on ACE activity, and NO generation and the inability of free radicals and PGs to suppress ACE activity are interesting since there is a close interaction between platelets, leukocytes and endothelial cells that may have relevance to their involvement in CVD (cardiovascular diseases). For instance, under normal conditions, endothelial cells produce adequate amounts of PGE_1 _from DGLA; PGI_2 _from AA; LXs and resolvins from AA, EPA and DHA; and NO from L-arginine such that the pro-inflammatory and pro-atherosclerotic events such as hemodynamic forces, hyperlipidemia, hypertension, smoking are successfully abrogated. These factors induce the expression of pro-inflammatory genes that initiate and accelerate atherosclerosis at the points of shear stress, enhance infiltration of intima by leukocytes and macrophages, cause low-level activation of NF-κB and elevated expression of VCAM-1 and ICAM-1, IL-1, IL-6, MCP-1, as well as antioxidant genes glutathione peroxidase and glutathione-S- transferase 2, and pro-inflammatory eicosanoids such as TXA_2_, PGE_2_, PGF_2α_, LTs, and other PGs, TXs, and LTs, and increased production and release of free radicals and UCP (uncoupling proteins) expression occurs in endothelial cells, platelets, and leukocytes in atherosclerosis-susceptible regions, and endothelial cells themselves may show changes in cell shape and proliferation. These events can be prevented and atherosclerosis process and the onset of CVD can be arrested if the production of PGE_1_, PGI_2_, PGI_3_, LXs, resolvins, NO, and anti-inflammatory cytokines such as IL-4, IL-10, TGF-β by endothelial cells is adequate, provided there are adequate stores of respective precursors of various PUFAs and L-arginine and their respective enzymes. This suggests that under physiological conditions a delicate balance is maintained between pro- and anti-inflammatory and pro and anti-atherosclerotic factors and when this balance is tilted more towards the former atherosclerosis and CVD occurs (reviewed in [80]).

In addition, these results suggest that when tissue concentrations of PUFAs are low, the activity of ACE will be high resulting in increased formation of angiotensin-II and a simultaneous decrease in eNO. In this context, it is important to note that transgenic rats overexpressing human renin and angiotensinogen genes (dTGR) develop hypertension, inflammation, and renal failure, and showed specific renal P450-dependent AA metabolism changes that led to decreased formation epoxy-eicosatrienoic acids (5,6-, 8,9-, 11,12- and 14,15-EETs) and hydroxyeicosa-tetraenoic acids (19- and 20-HETEs). Both EETs and HETEs inhibit IL-6 and TNF-α-induced activation of NF-κB and prevent vascular inflammation [81], suggesting that AA and other PUFAs not only regulate ACE activity and Ang-II levels in the tissues but also possess anti-inflammatory properties by generating anti-inflammatory metabolites.

AA, EPA, and DHA are converted in the presence of aspirin to epi-lipoxins, lipoxins, and resolvins that possess potent anti-inflammatory actions (reviewed in [18]). Epi-lipoxins enhance the formation of eNO [18,21,22]. NO blocks the interaction between leukocytes and the vascular endothelium and also stimulates the formation of PGI_2_, a potent vasodilator and platelet anti-aggregator, from AA [82]. This suggests that the beneficial actions of aspirin could be attributed not only to its ability to enhance the formation of PGI_2 _and suppress the synthesis of TXA_2 _but also to the formation of epi-lipoxins and eNO [20,21,82]. Thus, PUFAs regulate renin formation and action, inhibit angiotensin-II formation by its action on ACE activity, enhance eNO formation, and form precursors to beneficial biologically active molecules such as PGE_1 _(from DGLA), PGI_2 _(from AA), PGI_3 _(from EPA), lipoxins (from AA, EPA, and DHA), resolvins (from AA, EPA and DHA), protectins (from DHA), and 5,6-, 8,9-, 11,12- and 14,15-EETs and hydroxyeicosa-tetraenoic acids (19- and 20-HETEs) (from AA), and thus serve as endogenous regulators of vascular tone, platelet aggregation, and blood pressure.

### Effects on platelets and other hemostatic indices

Aspirin is effective in the prevention and treatment of acute myocardial infarction (AMI) and in the secondary prevention of CVD [83], though the efficacy of aspirin in women is not certain [16]. One of the important constituents of the "polypill" is aspirin (75 mg). Studies revealed that low dose aspirin not only reduced risk of heart disease, but also reduced the incidence of lung, colon, and breast cancer [84]. Aspirin inhibits nuclear factor NF-κB transcription, blocks prostaglandin (PG) and thromboxane (TX) synthesis (TXA > PGI_2_). Aspirin does not inhibit the production of proinflammatory mediators such as leukotrienes (LTs). Although blockage of PGs and TXs accounts for many of aspirin's pharmacologic properties, recent studies revealed that aspirin evokes the formation of 15-epi-LXA_4 _by the acetylated PGHS-2 [prostaglandin G/H synthase (cyclooxygenase)] and 5-lipoxygenase enzymes as a result of endothelial cell-leukocyte interactions [85]. LXA_4 _inhibits polymorphonuclear leukocyte transmigration, modulate adhesion to endothelial cells, and inhibit chemotaxis of PMN and eosinophils. Thus, many beneficial actions of aspirin could be attributed to the formation of LXA_4_. In this context, it is noteworthy that AA, EPA, and DHA when used in appropriate concentrations and ratio can reproduce many of the beneficial actions of aspirin.

Both EPA and DHA, when given orally, are rapidly incorporated into platelets and compete with AA for the 2-acyl position of membrane phospholipid and as substrate for the cyclo-oxygenase (CO) and lipoxygenase (LO) enzymes. As a result, when stimulated, such platelets produce less amounts of TXA_2 _and more of TXA_3 _that is less potent in inducing platelet aggregation and thrombosis [86]. Increased intake of fish oil, a rich source of EPA and DHA, produces a lower platelet count, less platelet aggregation, a longer bleeding time, higher urinary PGI_2 _metabolites, and lower concentrations of thromboxane metabolites compared to those who were on Western diet [87,88], effects that are similar to those of low-dose aspirin and qualify ω-3 EPA and DHA to be termed as an "endogenous aspirin". In general, though EPA and DHA do not have a very significant effect on blood lipids (except to lower plasma triglycerides and VLDL with no significant action on HDL-C levels), fibrinolysis and on the activity of plasminogen activity inhibitor-type-1 (PAI-1), still are effective in preventing overall mortality from CVD [18,21,80,89-94].

It is believed that production of pro-inflammatory eicosanoids from AA and/or decreased synthesis of anti-inflammatory and beneficial eicosanoids from EPA/DHA could predispose an individual to cardiovascular risk and stroke. Thus, it is thought that products of AA such as TXA_2_, PGEs, PGF_α_s, and LTs contribute or initiate the process of atherosclerosis in coronary arteries and cause CHD. In contrast, it has been proposed that beneficial products or less harmful products formed from EPA and DHA are less likely to cause atherosclerosis and CHD or even regress atheroma and prevent CHD. Although this is an attractive hypothesis, hard data in support of this proposal are not forthcoming. In this context, it should be noted that not all products of AA are harmful. For instance, PGI_2 _formed from AA is a potent vasodilator and platelet anti-aggregator and prevents atherosclerosis and has anti-arrhythmic action [95].

Similarly, DGLA, another ω-6 fatty acid that is the precursor of AA, gives rise to PGE_1_, which is a vasodilator, platelet anti-aggregator and anti-arrhythmic molecule [96,97]. These data emphasize the complexities involved in making generalizations about ascribing negative role to ω-6 fatty acids and their products in CVD. Furthermore, AA forms precursor to LXs and resolvins (See Figure 1) that have beneficial actions in resolving inflammation. In addition, Harris et al [98] noted that pooling of data from case-control or prospective cohort studies showed that none of the individual fatty acids computed across datasets were significantly different between cases and controls. EPA was 8.2% lower (p = 0.06), DHA was 8% lower in cases compared with controls, whereas DPA (docosapentaenoic acid) was virtually identical in both controls and CHD patients. In contrast and contrary to expectations, AA was 8.5% lower in cases compared to controls. The unexpected finding that AA concentrations were lower in patients with CHD suggests that it is the deficiency of AA rather than its excess that predisposes to CHD events. In addition, data from the Health Professionals' Follow-up Study [99] revealed that while there is an inverse relationship between ω-3 fatty acid intake and future risk for CHD, higher intakes of ω-6 fatty acids did not diminish the beneficial effects of ω-3 fatty acids suggesting that the absolute intakes of the ω-3 fatty acids are more important than the ratio between ω-3 fatty acids and ω-6 fatty acids. In this context, the interaction(s) between ω-3 and ω-6 fatty acids are significant.

### Interaction(s) between ω-3 and ω-6 fatty acids and its relevance to CHD/CVD

In a case control study of new angina pectoris and first acute myocardial infarction, a progressive inverse relation between adipose tissue LA and the estimated relative risk of CHD was noted [100]. Wood, *et al *[101] observed that low concentrations of DGLA in adipose tissue showed a more significant relation to new CHD than did LA. In an extension of this study, it was noted that there is a progressive inverse relations between adipose LA and platelet-membrane EPA and the estimated relative risk of angina pectoris. These relations were statistically independent of each other and traditional CHD risk factors [100-102].

Luostarinen, *et al *[103] noted that the percentage of palmitic acid and LA were significantly higher and the percentage of AA and of all the other major PUFAs, both ω-3 and ω-6, was significantly lower in the total phospholipid fraction of human coronary arteries of those who had sudden cardiac death due to CHD. Felton, *et al *[104] reported that the concentrations of all fatty acids were increased at the edge of disrupted plaques compared with the center, but as a proportion of total fatty acids, ω-6 were lower. These results suggest that ω-6 fatty acids have a significant role in CHD and it is likely that some of the inconsistent results obtained in some studies with EPA and DHA could be attributed to inadequate provision or utilization of ω-6 fatty acids, DGLA and AA. It is possible that there is a close interaction between ω-3 and ω-6 fatty acids, which could influence one's susceptibility or resistance to CHD. In this context, it is interesting to note that EPA/DHA readily get incorporated into the atheromatous plaque, and patients treated with fish oil had more thick fibrous caps and no signs of inflammation compared with plaques in patients in the control and sunflower oil groups. Furthermore, the number of macrophages in plaques from patients receiving fish oil was lower than in the other two groups, suggesting that atherosclerotic plaques readily incorporate ω-3 PUFAs from fish-oil supplementation, inducing changes that can enhance stability of atherosclerotic plaques [105].

Studies revealed that ω-3 and ω-6 fatty acids interact with each other in such a way that one potentiates the metabolism of the other. For instance, in perfused vascular tissue, DGLA increases the conversion of EPA to PGI_3_, a potent vasodilator and platelet anti-aggregator [106], whereas AA augmented the conversion of EPA to PGI_3 _in the tissues [107-109]. EPA inhibits the activity of the enzyme Δ^5 ^desaturase that results in an increase in the concentrations of DGLA in the tissues (especially in the endothelial cells). This increase in tissue levels of DGLA could enhance the formation of PGE_1_, a vasodilator and platelet anti-aggregator (see Figure 1). Thus, EPA can indirectly enhance the formation of PGE_1_. Furthermore, even the beneficial action of statins (HMG-CoA reductase inhibitors) and glitazones (PPARs agonists) seem to be mediated by EFAs and their metabolites such as LXs, resolvins, and neuroprotectins [28-33], [110-114], which are potent anti-inflammatory molecules [18,21,115-117]. Studies did suggest that ω-3 fatty acids decreased the levels of pro-inflammatory cytokines, and enhance that of IL-10, an anti-inflammatory cytokine [118-120]. This close interaction between ω-3 and ω-6 fatty acids and their ability to modify inflammatory markers, production of PGI_2_, PGE_1_, PGI_3_, LXs, resolvins, neuroprotectins, NO, nitrolipids, and the action of statins and glitazones on EFA metabolism and NO explains the relationship between various fatty acids and CHD and stroke (Figure 1).

### PUFAs in renal function

One of the components suggested to be included in the "polypill" is a thiazide, a diuretic and an anti-hypertensive drug. If PUFAs are to be considered to function as an endogenous "polypill", then they (PUFAs) should show beneficial actions on renal function.

Healthy volunteers given EPA (3.9 gm) and DHA (2.4 gm) per day for 6 weeks showed significant increase in renal plasma flow, glomerular filtration rate, decrease in renal vascular resistance, and an increase in excretion of PGE_3 _with no change in blood pressure and heart rate [121]. Diet rich in evening primrose oil (a rich source of GLA and LA) and safflower oil decreased proteinuria, glomerular sclerosis, and tubular abnormalities in diabetic rats, and showed increased ratio of renal cortical production of 6-keto-PGF_1α _(a metabolite of PGI_2_) to TXB_2 _with no significant changes in plasma lipid composition. In contrast, fish oil feeding decreased plasma lipids and lowered 6-keto-PGF_1α_/TXB_2 _ratio without any effect on renal disease in diabetic rats [122]. Singer et al [123] observed that spontaneously hypertensive rats had significantly lower systolic blood pressure when fed fish oil (EPA and DHA), evening primrose oil (a rich source of GLA), and fish oil + evening primrose oil, suggesting that a combination of GLA, EPA, and DHA produces optimal beneficial actions with regard to renal indices and blood pressure. Vaskonen et al [124] reported that fish oil prevented rise in blood pressure induced by high-salt diet in stroke-prone spontaneously hypertensive rats. This beneficial effect on blood pressure was associated with a decrease in TXB_2 _formation by 75% and an increase in plasma and renal ω-3 fatty acid content.

Furthermore, EPA/DHA suppressed mesangial cell proliferation, arrested progression of IgA nephropathy, and protected against cyclosporine-induced renal damage [125-127]. These results suggest availability of optimal amounts of GLA and EPA/DHA is necessary to reduce blood pressure and preserve renal function in diabetic and hypertensive rats. Studies performed with 5/6 renal ablation rat model that developed hypertension, albuminuria, and a decline in glomerular filtration rate had significantly less glomerulosclerosis and dyslipidemia when supplemented with fish oil and flax seed oil (rich in ALA) compared with the control group at 10 and 20 wk post-surgery [128,129]. Thus, PUFAs may show actions similar to those observed with conventional, synthetic diuretics. These beneficial actions of PUFAs can be attributed to the formation of beneficial PGA, PGE_3_, PGI_2_, PGI_3_, and recently identified resolvins and protectins and decrease in the production of TXA_2 _and LTs [130]. It is interesting that diuretic furosemide enhances endothelial synthesis and release of bradykinin and related kinins that, in turn, stimulates endothelial PGI_2 _formation via B2 kinin receptor activation [131] and COX-2 derived PGs interact with the renin-angiotensin system to regulate renal function [132].

### PUFAs and parasympathetic nervous system

Previous studies showed that multiple-blood pressure lowering drugs have additive effects, which led Wald and Law to suggest that a β-blocker need to be added to the "polypill" composition. This is in addition to the presence of a diuretic (such as a thiazide) and an ACE-inhibitor [11]. Autonomic function is an important factor that regulates heart rate, blood pressure and cardiac rhythm. Hence, it is expected that addition of β-blocker to the composition of "polypill" will reduce sympathetic tone, blood pressure and heart rate.

Autonomic function is assessed by the measurement of heart rate variability (HRV) and the evaluation of baroreflex sensitivity (BRS). HRV reflects the physiological levels of tonic autonomic regulation, whereas BRS indicates the capacity of reflex autonomic regulation. Both low HRV and low BRS are associated with increased cardiovascular risk. Vagal stimulation by a release of acetylcholine (ACh) and adrenergic stimulation mediated by norepinephrine and epinephrine regulate the autonomic function and thus the variations in HRV and BRS. Several studies revealed that ω-3 fatty acids reduce the risk of sudden death by preventing life-threatening cardiac arrhythmias and by significantly increasing HRV [133]. Furthermore, a direct positive correlation was noted between the content of DHA in cell membranes and HRV index suggesting an anti-arrhythmic effect of ω-3 fatty acids [134]. Since increased parasympathetic tone is responsible for increase in ventricular fibrillation threshold and protects against ventricular arrhythmias, it is likely that EPA/DHA supplementation enhances parasympathetic tone. This is supported b the observation that EPA/DHA supplementation increases hippocampal ACh levels, the principal neurotransmitter of parasympathetic nerves [135]. Hence, it is likely that EPA/DHA supplementation increases the brain ACh levels leading to an increase in the parasympathetic tone and so an increase in HRV and protection from ventricular arrhythmias. Similar to EPA/DHA, AA also augments ACh release [136,137], and thus, PUFAs enhance parasympathetic tone resulting in an increase HRV and prevention of ventricular arrhythmias.

Vagus nerve stimulation also inhibits TNF synthesis in liver and ACh significantly attenuated the release of pro-inflammatory cytokines: TNF-α, IL-16, IL-1β, and IL-18 but not anti-inflammatory cytokine IL-10 by stimulated macrophages *in vitro *and *in vivo *[138-140]. Thus, one mechanism by which PUFAs suppress inflammation could be by augmenting the release of ACh and enhancing the parasympathetic tone.

Since, normally a balance is maintained between parasympathetic and sympathetic tones, it is reasonable to suggest that whenever parasympathetic tone (vagal tone) is enhanced sympathetic tone is reduced (akin to blocking of β-receptors as it occurs in instances of use of β-blockers). Thus, indirectly PUFAs may function like β-blockers.

### Folic acid and PUFAs

Hyperhomocysteinemia is a risk factor for cardiovascular diseases, and it may interact with hypertension and an unfavorable cholesterol profile to alter the risk of CVD. Hence, folic acid (0.8 mg/day) has been added as a component of the "polypill". It is important to note that folic acid increases concentrations of ω-3 PUFAs which could reduce the risk of thrombosis and CVD [141-143]. It was observed that some of the adverse effects induced by folic acid deficiency could be overcome by supplementing with ω-3 EPA and DHA [144], and in folic acid deficiency states the plasma and brain concentrations of PUFAs are decreased [145]. These results imply that some, if not all, actions of folic acid are mediated by PUFAs.

Thus, PUFAs, when given in appropriate dose and combination (containing EPA, DHA and possibly, GLA, DGLA and AA) show all the qualities of the suggested "polypill", viz., aspirin-like action, inhibit the activities of HMG-CoA and ACE enzymes, possess diuretic and anti-hypertensive actions, and indirectly show β-blocker-like action. In addition to these useful actions, PUFAs also have other beneficial actions as described below.

### PUFAs inhibit cholesteryl ester transfer protein (CETP) activity

HDL-cholesterol (high-density lipoprotein- cholesterol, HDL-C) is an independent risk factor for CHD. Higher plasma HDL-C is associated with a decreased incidence of CHD [146] that led to the suggestion that therapeutic strategies that raise HDL-C could be of benefit in preventing CHD by increasing the movement of cholesterol from the periphery back to the liver (the so-called reverse cholesterol transport or RCT pathway).

CETP is a hydrophobic plasma glycoprotein, mainly synthesized in the liver, possessing the unique ability to facilitate the transfer of cholesteryl ester (CE). CETP circulates in the blood, bound predominantly to HDL. CETP mediates the transfer of cholesteryl esters from HDL to VLDL and LDL in exchange for triglycerides and promotes the transformation of HDL_2 _to HDL_3_, an action that could promote reverse cholesterol transport. CETP inhibition produces an increase in HDL by markedly delaying catabolism of apoA-I and A-II [147], an action that increases reverse cholesterol transport. These actions suggest that CETP inhibition could prevent atherosclerosis and prevent CHD [148-150].

In healthy, normolipidemic men, it was observed that lipid-lowering diet rich in monounsaturated fatty acid (oleic acid) decreased CETP concentrations to a significant degree [151]. In HepG2 cells, 0.5 mM of AA, EPA, and DHA reduced the levels of CETP mRNA by more than 50% of the control levels with a corresponding significant decrease in the CETP mass [152]. A significant negative correlation was found between plasma CETP activity and monounsaturated fatty acid content of plasma phospholipids or free PUFAs including ω-3 fatty acids, suggesting that PUFAs suppress CETP activity [153].

Torcetrapib, a small molecule inhibitor of CETP, is very effective at raising HDL-C and apolipoprotein A-I and decreasing levels of LDL-C and apolipoprotein-B-100 and also showed favourable effects on increasing the size of HDL-and LDL particles. In patients with familial hypercholesterolemia, torcetrapib with atorvastatin as compared with atorvastatin alone did not result in reduction of progression of atheroslcerosis as measured by carotid arterial-wall thickness despite a significant increase in HDL-C levels and decrease in levels of LDL-C and triglycerides. In fact, it was observed that administration of torcetrapib with atorvastatin was associated with progression of atherosclerosis [154], and an increase in blood pressure with no significant decrease in the progression of coronary atherosclerosis [155]. These results with torcetrapib + atorvastatin suggest that simultaneous inhibition of CETP and HMG-CoA reductase enzyme leads to an elevation of plasma HDL-C, and decrease in LDL-C and triglycerides and cholesterol but it does not arrest progression of atherosclerosis. In contrast, PUFAs, especially ω-EPA and DHA, not only inhibit CETP and HMG-CoA reductase enzyme, lower plasma triglycerides, cholesterol, and LDL-C with little or no change in HDL-C but also are effective in arresting atherosclerosis and preventing CHD [156-165]. In contrast to the results with torcetrapib + atorvastatin, Yokoyama *et al *[166] reported that a combination of ethyl EPA + 10 mg of pravstatin or 5 mg of simvastatin prevented major coronary events and especially non-fatal coronary events in Japanese hypercholesterolemic patients with a mean period of follow up of 4.6 years. It is interesting to note that the benefits were in addition to statin treatment, and fish oil was found to be safe and well tolerated. These results once again confirm that EPA and DHA are of benefit in the prevention and treatment of cardiovascular diseases. Thus, PUFAs appear to be superior to CETP and statins in the prevention of CVD despite the fact that they do not necessarily increase plasma HDL-C levels.

### EPA, DHA, and PGI2 function as endogenous anti-arrhythmic molecules

There is reasonable evidence to suggest that EPA/DHA and PGI_2 _have anti-arrhythmogenic effects. Various PUFAs and PGs are present in the heart including SA (sinoatrial) node [167,168]. I showed that PGE_1_, PGE_2_, PGI_2_, and TXB_2 _modify contractions of isolated rat cardiac muscle cells and increased the amount of ^45^Ca^2+ ^exchanged by non-beating cells [169]. These results led to the suggestion that PGI_2 _could be an endogenous anti-arrhythmic molecule [170]. It was reported that PUFAs increased the electrical threshold for the induction of ventricular fibrillation that could reduce the risk of developing malignant cardiac arrhythmias. Mitochondrial dysfunction induced by EFA deficiency could be eliminated by the presence of normal levels of the essential fatty acids in the ω-3-enriched mitochondrial membrane phospholipids [171] that may account for the ability of EPA/DHA to decrease cardiac arrhythmias during myocardial ischemia. The recovery of mitochondrial energy metabolism and myocardial pump function during reperfusion is significantly better in ω-3 PUFA-enriched hearts, suggesting that EPA and DHA limit myocardial injury during ischemia and reperfusion [172].

EPA and DHA (at 2–10 microM) reduced the contraction rate of spontaneously beating, isolated, neonatal rat cardiac myocytes [173] without a significant change in the amplitude of the contractions. Both CO- and LO-inhibitors and antioxidants did not alter the effect of the fatty acids. The inhibitory effect of EPA and DHA on the contraction rate was similar to that produced by the class I antiarrhythmic drugs. It was also reported that lysophosphatidylcholine (LPC)- or acylcarnitine-induced arrhythmias were completely blocked by EPA, DHA, ALA, AA, and LA by inhibiting the electrical automaticity/excitability of the cardiac myocytes. Studies using whole-cell patch-clamp technique in cultured neonatal rat ventricular myocytes revealed that EPA, DHA, and to a limited extent AA can produce a concentration dependent suppression of ventricular, voltage-activated Na^+ ^currents that may explain their anti-arrhythmic actions in vitro and in vivo [174-176].

### PUFAs modulate telomere and telomerase activity

Telomere, the genetic segment that appears at the end of the chromosomes, has the special property of protecting these ends. Telomerase is the enzyme that adds telomere repeats to the ends of the chromosomes with the use of a dedicated RNA template. Inactivation of telomerase leads to telomere shortening and eventual senescence of the cells. Telomerase consists of two principal subunits: telomerase reverse transcriptase (TERT), the protein catalytic subunit, and the telomerase RNA component (TERC). Primary cells when grown in vitro, lack sufficient TERT to maintain telomeres and hence, telomeres shorten progressively with each cell division. This eventually results in shorter telomere that loses its ability to protect the ends of chromosomes and is therefore recognized by the cell's DNA repair machinery as damaged DNA. The loss of telomere results in cellular senescence since cell can no longer divide and replicate itself. In contrast, overexpression of TERT prevents telomere attrition and enables cells to proliferate indefinitely, a character of cancer cells. Thus, telomere and telomerase are central to several diseases such as cancer, aging, atherosclerosis, CHD, type 2 diabetes, hypertension, and to the biology of stem cells.

Recent reports suggested that leukocyte telomere length is a predictor of future CHD in middle-aged, high-risk men, whereas 10 mg of pravastatin, a HMG-CoA reductase inhibitor, substantially abrogated shortening of the telomere length in high-risk subjects [177-179], suggesting that patients with CHD have senescent endothelial cells. Telomere shortening has been reported in patients with type 2 diabetes mellitus, hypertension, and insulin resistance [180-184]. Diabetes mellitus, hypertension, insulin resistance, and CHD are not only low-grade systemic inflammatory conditions in which plasma levels of lipid peroxides, IL-6, and TNF-α are increased and eNO and concentrations of anti-oxidants are decreased, but also show shorter leukocyte telomere length compared to controls. NO activates telomerase [185] and delays endothelial cell senescence [186], whereas asymmetrical dimethyl arginine, an inhibitor of NO synthesis, enhances endothelial cell senescence [187,188]. It was reported that stable expression of hTERT (human telomerase reverse transcriptase) enhances production of eNO and NO activity and renders endothelial cells to show younger phenotype [189,190], whereas NO activates telomerase and delays endothelial cell senescence. These results imply that NO prevents whereas reactive oxygen species induce telomere shortening.

In contrast, tumor cells express increased telomerase activity. Tumor cells have relatively higher content of anti-oxidants and reduced concentrations of lipid peroxides due to PUFA deficiency. It is known that ω-3 PUFAs are of benefit in type 2 diabetes, hypertension, and hypertriglyceridemia and prevent CHD, in part, by enhancing NO generation from endothelial cells and decreasing insulin resistance [191]. Tumor cells undergo apoptosis on exposure to ω-3 PUFAs (especially in response to EPA, DHA, and GLA) due to increase in intracellular free radical generation and formation of lipid peroxides. Since, NO and lipid peroxides modify telomerase activity, it is likely that PUFAs enhance or decrease activity of TERT in endothelial cells and tumor cells respectively. This is supported by the observation that EPA and DHA inhibit hTERT activity in human colorectal adenocarcinoma cells [192,193]. Thus, PUFAs can prevent, reverse or arrest atherosclerosis and CHD by their ability to enhance eNO synthesis that, in turn, augments hTERT activity and prevents endothelial senescence.

## Conclusion

It is evident from the preceding discussion that PUFAs, especially an optimal combination of EPA, DHA and possibly, GLA, DGLA and AA show most the qualities of the suggested "polypill", viz., aspirin-like action, inhibition of HMG-CoA and ACE enzymes, and possess diuretic, anti-hypertensive, and β-blocker-like actions (see Table 1 for a summary of actions). PUFAs are naturally occurring endogenous substances, present in almost all tissues and are essential components of all mammalian cells and have been shown to be relatively safe when administered to different types of patients for long periods of time (from few months to few years). This is evident from the fact that Eskimos consume large amounts of marine fish that are rich in ω-3 fatty EPA and DHA and are not known to suffer from any significant side effects. Nevertheless, possible side effects due to long-term feeding of PUFAs need to be studied. One concern that is generally expressed about PUFAs is that their increased intake may enhance lipid peroxidation, and that these oxidized products could be harmful to tissues. But, it was reported that increased intake of EPA/DHA, in fact, reduces in vivo lipid peroxidation and oxidative stress in humans [194-196]. Furthermore, peripheral leukocytes, which are major mediators of inflammation, are capable of *de novo *production of catecholamines that enhance the inflammatory response [197]; whereas vagal parasympathetic signaling suppresses inflammation through cholinergic receptors on these cells [138]. This suggests that sympathetic and parasympathetic pathways and immune system cross talk with each other during inflammation. PUFAs, especially ω-3 fatty acids enhance acetylcholine levels [135-137] and increase HRV [133,134] due to their ability to augment parasympathetic tone and thus, indirectly function as endogenous suppressors of sympathetic nervous system and thus, of β-receptor function. This is especially so since under normal conditions a balance is maintained between sympathetic and parasympathetic systems and pro- and anti-inflammatory pathways. These evidences imply that PUFAs function as endogenous enhancers of parasympathetic tone, suppress inflammatory events; and inhibit sympathetic over activity, and block β-receptor action.

**Table 1:** Possible cumulative impact of four secondary prevention treatments in the prevention of cardiovascular diseases.

Drug therapy	Relative-risk reduction	2-year event ratio
None	-	8%
Aspirin	25%	6%
B-blockers	25%	4.5%
Lipid lowering (by 1–5 mmol)	30%	3.0%
ACE inhibitors	25%	2.3%

Cumulative relative reduction if all four drugs are used is about 75% [see ref. [91]].

Events that were included in this analysis are: cardiovascular death, myocardial infarction or strokes.

**Table 2:** Actions of PUFAs (especially of ω-3 fatty acids) that account for their beneficial actions in inflammation, atherosclerosis, hypertension, hyperlipidemias, type 2 diabetes mellitus and coronary heart disease.

Target	Effect
Plasma triglyceride concentration-fasting and post-prandial	↓↓
Plasma cholesterol	↓↔
HDL cholesterol	↑↔
LDL cholesterol	↓↔
Blood pressure	↓
Diuretic-like action	↑
Endothelial production of NO	↑
ACE activity	↓
HMG-CoA activity	↓
Platelet aggregation	↓
Leukocyte activation	↓
Cardiac arrhythmias	↓
Heart rate variability	↑
Production of lipoxins and resolvins	↑
Formation of lipid peroxides	↓
Production of PGI_2_, PGI_3_, PGE_1_	↑
Production of TXA_2_, LTs	↓
Synthesis of pro-inflammatory cytokines such as TNF-α and MIF	↓
Production of anti-inflammatory cytokines such as IL-10	↑
Insulin sensitivity	↑
Endothelial integrity	↑
Telomere length	↑
Parasympathetic tone	↑
Sympathetic tone	↓

In view of these beneficial actions (see Table 2 for a summary of their actions), ω-3 and ω-6 fatty acids can be given for the prevention of CVD. Since PUFAs can be given to pregnant women and lactating mothers, and children, it is suggested that a combined ω-6 and ω-3 pill could be given from childhood. Furthermore, several studies suggested that PUFAs, especially ω-3 fatty acids, are useful in the prevention and treatment of Alzheimer' disease, schizophrenia, and depression [198-209], suggesting that PUFAs have a much wider benefit compared to the "polypill". It may also be mentioned here that for their physiological/beneficial action(s) PUFAs need many co-factors such as folic acid, vitamin B_12_, vitamin B_6_, vitamin C, tetrahydrobiopterin (H_4_B), zinc, magnesium, calcium, L-arginine, and small amounts of selenium and vitamin E [18]. Hence, it is essential that these co-factors should also be provided in adequate amounts to bring about the beneficial action of ω-6 and ω-3 PUFAs. Since statins, glitazones, several anti-hypertensive and anti-arrhythmic drugs seem to be mediate their actions by modulating EFA/PUFA metabolism, it is possible that sub-clinical deficiency or altered metabolism of EFAs may subvert their actions/benefits. Hence, it is prudent to provide a combination of ω-6 and ω-3 PUFAs and their co-factors along with statins, glitazones, and other drugs in the treatment of CVD. Yokoyama et al [166] showed that long-term use of ethyl EPA (1800 mg/day) produced a significant reduction in non-fatal coronary events in patients with dyslipidemia compared to control. This risk reduction occurred after 2.5 years of use of ethyl EPA (mean follow up period was 4.6 years) even when PUFAs were added in addition to statin treatment lending support to the concept that ω-3 and ω-6 PUFAs can be combined with other cardiovascular drugs in the prevention and treatment of cardiovascular diseases.

## Competing interests  

UND owns and runs the biotech company UND Life Sciences that specialises in developing lipid-based drugs for cancer, diabetes melltus and hypertension.

## Author's Contributions 

I contributed to everything.
